# Translucency of 3D‐Printed 3Y‐ and 5Y‐Doped Zirconia

**DOI:** 10.1111/jerd.70072

**Published:** 2025-12-09

**Authors:** Sebastian Hetzler, Clemens Schmitt, Peter Rammelsberg, Stefan Rues, Andreas Zenthöfer

**Affiliations:** ^1^ Department of Prosthodontics Medical Faculty, Heidelberg University Heidelberg Germany

**Keywords:** 3D printing, dental zirconia, digital light processing, translucency, zirconia

## Abstract

**Objectives:**

To evaluate and compare the translucency of 3D‐printed zirconia materials: 3Y tetragonal zirconia polycrystal (3Y‐TZP) and 5Y partially stabilized zirconia (5Y‐PSZ).

**Material and Methods:**

3D‐printed 5Y‐PSZ and 3Y‐TZP materials fabricated using digital light processing (DLP) were investigated. In addition, three zirconia materials made for the milling approach were examined: one 3Y‐TZP and two 5Y‐PSZ variants. For translucency measurements, samples with a square base of 100 mm^2^ and five different thicknesses ranging from 0.25 to 1.25 mm (±50 μm tolerance) were produced (*n* ≥ 5/subgroup). The L*a*b* color coordinates were measured using an experimental setup consisting of a spectroradiometer, an integrating sphere, and a 35‐W tungsten lamp. The translucency parameter was calculated using the color difference between white and black backgrounds. The microstructure and grain size were analyzed using scanning electron microscopy.

**Results:**

Translucency increased with a decrease in thickness, across all materials. Milled materials exhibited higher translucency than 3D‐printed counterparts with equivalent yttria content. The 3D‐printed 5Y‐PSZ and milled 3Y‐TZP materials displayed comparable translucencies. Microstructural analysis revealed discrete microporosities in the 3D‐printed materials.

**Conclusions:**

Despite microstructural differences and defects reducing the translucency of 3D‐printed zirconia compared to their milled counterparts, 3D‐printed 5Y‐PSZ demonstrates sufficient optical properties for monolithic restorations in posterior regions.

## 1 Introduction

Zirconia‐based materials are widely used in many dental restorations [1, 2, 3, 4]. This has been driven particularly by the further development of high‐strength, tooth‐colored, and biocompatible materials with regard to translucency [5, 6]. Zirconia restorations are predominantly manufactured using a computer‐aided design and computer‐aided manufacturing (CAD/CAM) workflow involving milling based on CAD. The development of fully or partially stabilized zirconia with better esthetics compared to tetragonal zirconia polycrystal materials with 3 mol% yttria content (3Y‐TZP) by doping with higher yttria contents (4–5 mol%) and zirconia blanks for milling with color and/or strength and translucency gradients has expanded its indications. This enabled the monolithic use without the need for veneering in specific clinical situations, at least in the posterior region and/or mandibula [7, 8, 9]. Glass ceramics ideally mimic the optical properties of natural tooth structure due to their fine microstructure of a glassy matrix and embedded crystals with refractive indices close to enamel and dentin [10, 11]. In contrast, zirconia is composed of polycrystals whose phase composition (tetragonal, cubic and monoclinic) depends on the yttria content, which also determines grain size and thus its translucency [12]. In addition, previous studies indicated that aging even increases the translucency of zirconia materials due to the alteration of grain composition [13]. However, advanced translucency of these materials is purchased with a decrease in strength. Adaptation of color and translucency is always a key task for a dental restoration with ideal natural appearances and is especially challenging in the anterior region if only one tooth should be rehabilitated. Specific characteristics of neighboring teeth like cracks or extra translucent areas can be more sufficiently imitated by the use of labial glass ceramic veneering of zirconia frameworks. In posterior regions full coverage restorations are more uncritical in adaptation; however, strictly defect‐oriented or partially covering restorations such as occlusal veneers are demanding in order to esthetically adapt the junction tooth/restoration. However, primarily in those posterior cases, the advantaged material properties of zirconia over other ceramics are a key benefit. The 3D zirconia printing is now an alternative to the classic milling process [14, 15, 16]. The advantages of additive manufacturing are evident, with higher design freedom and less waste production due to residual blank parts. Despite the emergence of clinically approved printing systems, the 3D printing of zirconia remains a niche technique. However, disadvantages such as the technically demanding preparation of printing suspensions consisting of acrylate binder and zirconia filings, as well as time‐consuming cleaning and extended debinding and sintering times, make the application less attractive than milling. Some studies that have examined material parameters, such as the strength of 3D‐printed standard test specimens or restorations made of 3Y‐TZP, have shown that the strength values and reliability are somewhat lower than those of their milled counterparts, although they are clinically sufficient [17, 18, 19, 20, 21]. Moreover, microstructural defects that preferentially align along the interfaces between different layers lead to variability in flexural strength depending on the nesting orientation of the object.

The fit of anterior veneers produced using 3D‐printing technology appears to be comparable to those fabricated with the established milling technology [22, 23, 24]. The industry has made continuous improvements in the 3D‐printing area. More translucent zirconia materials for zirconia printing such as partially stabilized zirconia doped with 5 mol% yttria (5Y‐PSZ), are now commercially available. The initial results indicate that this material has similar strength to milled 5Y‐PSZ, with possibly improved translucency compared to additively manufactured 3Y‐TZP, which justifies its use in the posterior regions in a monolithic design [19].

Therefore, this study aimed to investigate the translucency, measured by the translucency parameter, of 3D‐printed 5Y‐PSZ and 3Y‐TZP specimens compared to their milled counterparts. Based on previous findings in the literature [14, 15, 25] including our prior investigations [18, 19, 26], we hypothesized that (1) 3D‐printed zirconia would exhibit lower translucency than milled zirconia of similar yttria content, owing to the presence of microporosities inherent to the layer‐by‐layer additive manufacturing process and as previously observed in microstructural analyses of printed specimens, and (2) that 5Y‐PSZ would demonstrate higher translucency than 3Y‐TZP due to its higher cubic phase content and larger mean grain size.

## 2 Materials and Methods

### 2.1 Sampling

Two types of 3D‐printed zirconia (PZ) and three types of milled zirconia (MZ) were investigated. An overview of the respective material names and manufacturers is presented in Table 1.

**TABLE 1:** Overview of the materials under investigation during this study.

	3D‐printed Zirconia		Milled Zirconia		
	3Y‐TZP	5Y‐PSZ	3Y‐TZP	5Y‐PSZ	
Material name	InniCera BCM W1000	InniCera BCM W1000‐T	Cercon ht	Cercon xt	VITA YZ XT
Manufacturer	AON Seoul Republic of Korea		Dentsply Sirona Bensheim, Germany		VITA Zahnfabrik Bad Säckingen, Germany
CAM device	ZIPRO‐D (AON)	ZIPRO (AON)	Cercon brain expert (Dentsply Sirona)		
Abbreviation	3Y‐PZ	5Y‐PZ	3Y‐MZ	5Y‐MZ‐1	5Y‐MZ‐2

**FIGURE 1:**
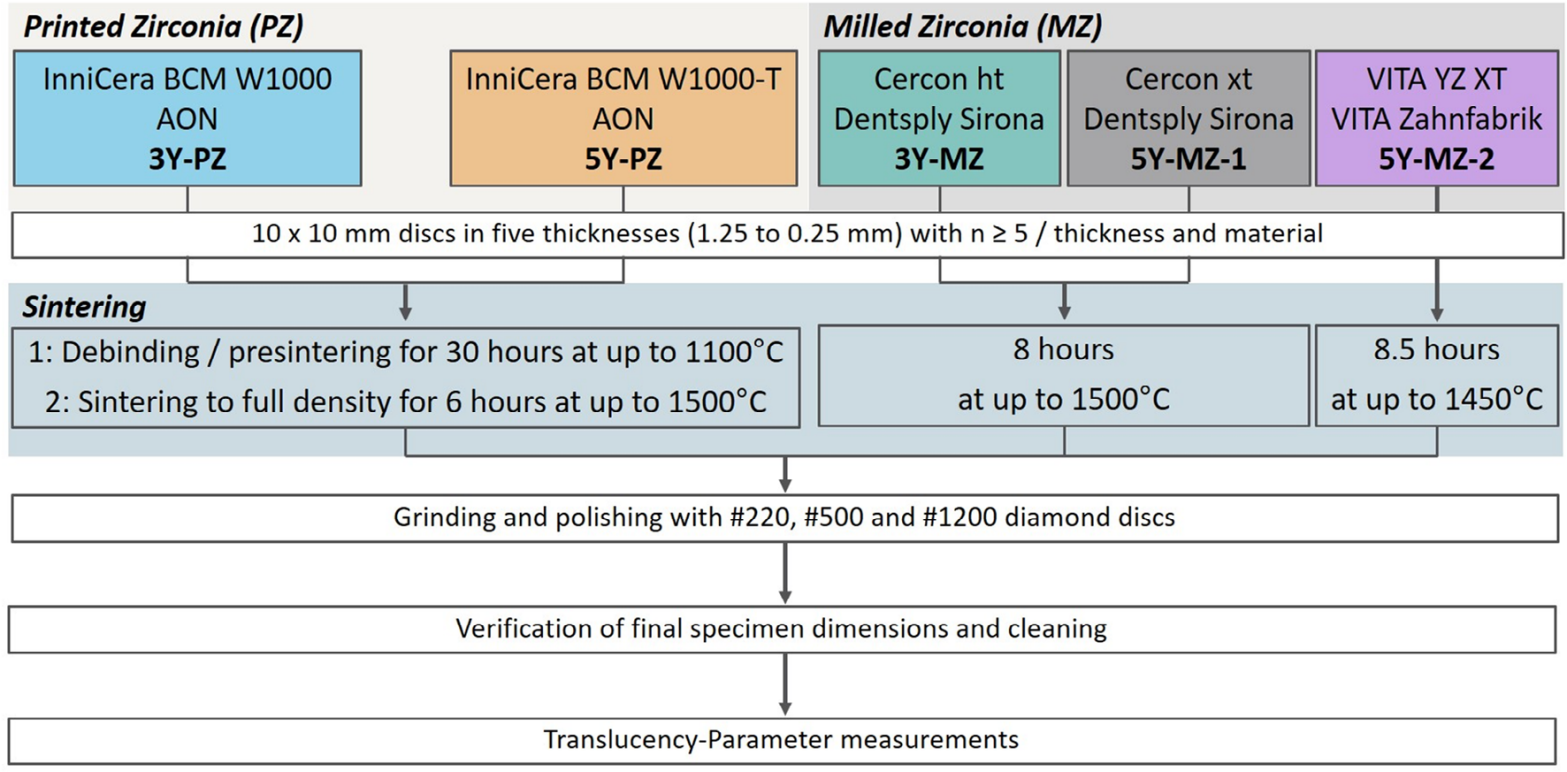
Sampling and study workflow.

Sampling and study workflow is visualized in Figure 1.

### 2.2 Sample Fabrication

Square disks with edge lengths of 10 mm were designed with different heights corresponding to nominal thicknesses of 0.25, 0.50, 0.75, 1.00, and 1.25 mm. A minimum sample size of *n* = 5 was used for each thickness and material type. This determination was based on observed mean differences and standard deviations of related studies in the literature [9, 27, 28]. To accommodate material removal during grinding and surface finishing, all specimens were initially designed and fabricated with an additional 0.40 mm thickness (i.e., specimen thicknesses of 0.65, 0.90, 1.15, 1.40, and 1.65 mm after sintering) using CAD software (Geomagic DesignX; 3D Systems, Rock Hill, SC, USA), and exported in surface tessellation language files.

The specimens intended for 3D printing (3Y‐PZ and 5Y‐PZ) were imported into the slicing software (ZiproS Version 4.1, AON), where they were oriented horizontally relative to the build platform and scaled accordingly. Support structures were added, as recommended by the manufacturer. The 3Y‐PZ specimens were printed using a Zipro‐D dental printer, whereas the 5Y‐PZ specimens were fabricated using the Zipro Dental printer (both AON). Both systems utilize a 405 nm light source and a layer thickness of 50 μm. No adjustments were made to the printing parameters between the two machines. After detachment from the build platform, the printed specimens were cleaned with isopropanol (purity ≥ 99.5%) using an airbrush system. Debinding and presintering were performed at temperatures up to 1100°C for 30 h 05 min (ZIRFUR, AON), followed by final sintering at a peak temperature of 1500°C for 6 h (SINTRA PRO/120zrf, Shenpaz Dental Ltd., Migdal HaEmek, Israel).

The specimens designated for milling (3Y‐MZ, 5Y‐MZ‐1, and 5Y‐MZ‐2; Table 1) were manufactured from commercially available zirconia blanks by using the same milling system (Cercon Brain Expert, Dentsply Sirona). Sintering was performed according to the respective manufacturer's specifications: 3Y‐MZ and 5Y‐MZ‐1 were sintered at a maximum temperature of 1500°C for 8.5 h (Cercon heat plus, Dentsply Sirona), while 5Y‐MZ‐2 was sintered at up to 1450°C for 8 h (Programat S1 1600, Ivoclar Vivadent).

Subsequent grinding and polishing of the final thickness were conducted in accordance with the ISO 6872 guidelines using diamond discs (MD Piano, #220, #500, and #1200; Struers, Willich, Germany) in a semiautomatic grinding and polishing device (Tegramin‐25, Struers). To verify the final specimen dimensions, three width measurements were performed for each sample using a digital micrometer screw (MicroMar 40 EWR; Mahr, Göttingen, Germany) and the values were averaged. Samples had to meet the nominal thickness with a tolerance of ±50 μm; otherwise, they were not included. Finally, all samples were manually cleaned with isopropanol (purity ≥ 99.5%).

### 2.3 Translucency Measurements

**FIGURE 2:**
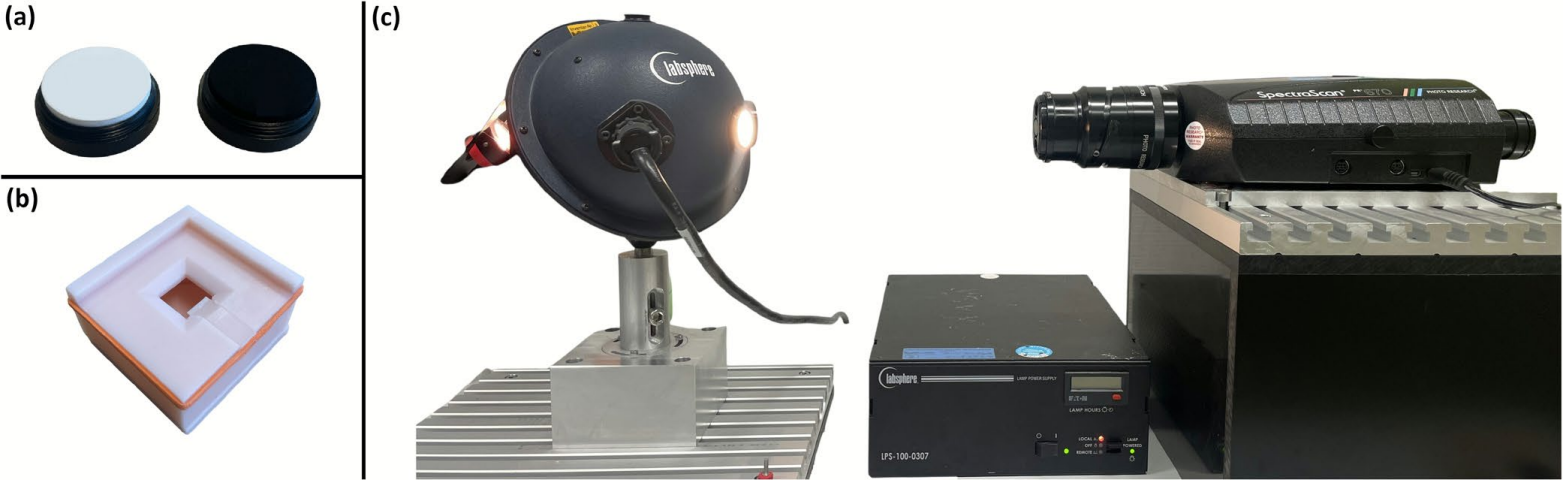
Measuring apparatus for determining the Translucency Parameter (TP). (a) Standardized white and black backgrounds (Labsphere North Sutton, NH), (b) Custom sample holder, and (c) Measuring device consisting of an Ulbricht sphere, a spectroradiometer, and a constant power supply.

The translucencies of the specimens were assessed by calculating the translucency parameter (TP). Color data were recorded according to the Commission Internationale de l'Éclairage (CIE) L*a*b* standard, which represents color as a specific point in a three‐dimensional color space. The measured color valences were given as L*a*b* coordinates with reference to the standard illuminant D 65 and were obtained at the center of the specimen and three times per specimen. The average value was used for the TP calculations. Measurements were conducted against standardized white (99% reflectance) and black (2% reflectance) backgrounds (Spectralon Diffuse Reflectance Standards SRS‐99‐010 and SRS‐02‐010; Figure 2a, Labsphere North Sutton, NH, USA) using a custom 3D‐printed specimen holder (Figure 2b). The TP value of each sample was determined using the CIELAB color difference formula (TP_*ab*_).

(1) $${\mathrm{TP}}_{\mathrm{ab}}=\sqrt{{\left(L_{b}^{*}-L_{w}^{*}\right)}^{2}+{\left(a_{b}^{*}-a_{w}^{*}\right)}^{2}+{\left(b_{b}^{*}-b_{w}^{*}\right)}^{2}}$$

Additionally, the CIEDE2000 (1:1:1) color difference formula was used to calculate the TP (TP_00_).

(2) $${\mathrm{TP}}_{00}=\sqrt{{\left(\frac{L_{b}^{*}-L_{w}^{*}}{k_{L}S_{L}}\right)}^{2}+{\left(\frac{C_{b}^{*}-C_{w}^{*}}{k_{C}S_{C}}\right)}^{2}+{\left(\frac{H_{b}^{*}-H_{w}^{*}}{k_{H}S_{H}}\right)}^{2}+R_{T}\left(\frac{C_{b}^{*}-C_{w}^{*}}{k_{C}S_{C}}\right)\left(\frac{H_{b}^{*}-H_{w}^{*}}{k_{H}S_{H}}\right)}$$

with subscript letters indicating L*, a*, and b* values against standardized white (w) and black (b) backgrounds. The weighting functions (*S*_L_, *S*_C_, *S*_H_) adjust the total color difference according to the location of the color pair in the L*, a*, and b* coordinates, while the rotation function (*R*_T_) accounts for the interaction between chroma and hue differences in the blue region. The parametric factors (*k*_L_, *k*_C_, *k*_H_), which correct for experimental conditions, were all set to 1 in the present study. The mathematical definition of these functions and parameters are pointed out by Sharma et al. [29].

A standardized experimental setup consisting of an Ulbricht sphere, a spectroradiometer, and a constant power supply unit was employed, as illustrated in Figure 2c. The spectroradiometer (PR‐670; SpectraScan, Photo Research, Chatsworth, CA) was equipped with a Macro‐Spectar MS‐75 lens (Photo Research) and positioned 400 mm from the sample. The aperture of the device was adjusted to 0.5°. The Ulbricht sphere provided illumination with a correlated color temperature of 6500 K and a color‐rendering index of 91. A regulated power supply (LPS‐100‐0307, Labsphere) was utilized to minimize fluctuations in the light output that affect the measurement accuracy.

To reduce light scattering, refraction, or reflection at the interfaces, a drop of glycerin (refractive index: 1.47) was applied between the background and the specimen, and all measurements were performed in a room without any further light sources.

### 2.4 Microstructural Analysis

Subsequent to the TP measurements for 3Y‐PZ and 3Y‐MZ, microstructural analysis and grain size determination were performed on selected specimens with a width of 1.25 mm. For all other materials, existing data on the microstructure were obtained from a previous study [19] on biaxial flexural strength using cylindrical specimens (12.4 mm in diameter and 1.2 mm in width). Analogous to this study, four specimens were randomly selected and each of the two regions of interest was evaluated. For this purpose, specimens received an additional mirror finish using diamond suspensions and polishing cloths in two steps (1: Diapro Dac 3 μm on MD Dac, 2: Diapro Nap B 1 μm on MD Nap; Struers), followed by thermal etching for 30 min at 300°C below the respective sintering temperature. Scanning electron microscope (SEM) images (JSM‐6510, JEOL, Freising, Germany) were obtained at various magnifications after gold sputtering (Cressington 108auto, Cressington Scientific Instruments Ltd., Watford, United Kingdom). The grain size was determined by a single operator using the line intersection method at 7500× magnification.

### 2.5 Statistical Analysis

All statistical analyses were performed using the SPSS Ver. 28 (IBM; New York, NY, USA). The level for local statistical significance was set to *α* = 0.05. The mean and standard deviation (SD) of the measured TP was calculated for each thickness of the different materials, and comparisons among the materials were performed for equal thicknesses. Individual test group data were normally distributed (Shapiro–Wilk tests), and equality of variances between groups could be assumed (Levene's test based on median: *p* = 0.277). This finding was reinforced by the acceptance of the null hypothesis of the Breusch‐Pagan heteroscedasticity test (the variance of the errors does not depend on the values of the variables, *p* = 0.585). Therefore, a two‐way ANOVA was conducted, followed by Tukey's HSD pairwise post hoc tests for overall comparisons between the materials. In addition, pairwise comparisons of the estimated marginal means (material × thickness) with Bonferroni adjustment were performed to examine material differences within each thickness level.

One‐way ANOVA and Tukey‐HSD post hoc tests were performed to evaluate the differences in grain size between all groups of PZ and MZ samples.

## 3 Results

### 3.1 Translucency

**FIGURE 3:**
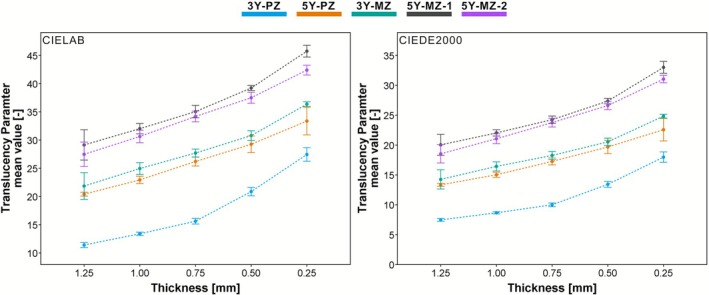
Mean translucency parameter over sample thickness for each material calculated using CIELAB (left) and CIEDE2000 (right). Whiskers refer to the 95% confidence interval.

The TPs of the different groups are presented in Tables 2 and 3 and Figure 3. Statistical analysis revealed that the material type and thickness significantly affected the TP (*p* < 0.001). As expected, TP increased with decreasing thickness for all the investigated materials. However, the impact of the change in thickness on the TP differed between the materials (*p* < 0.001). This is illustrated by the different slopes of the curves for each material in the TP‐thickness diagram, as depicted in Figure 3.

**TABLE 2:** Mean translucency parameter of the investigated materials across different wall thicknesses calculated using CIELAB.

Material	Specimen thickness [mm]				
	1.25	1.00	0.75	0.50	0.25
3Y‐PZ	11.41 (0.35)^A^	13.40 (0.23)^A^	15.63 (0.48)^A^	20.87 (0.60)^A^	27.45 (0.96)^A^
5Y‐PZ	20.41 (0.32)^B^	22.95 (0.61)^B^	26.21 (0.76)^B^	29.26 (1.39)^B^	33.37 (1.53)^B^
3Y‐MZ	21.86 (1.91)^B^	24.96 (0.84)^C^	27.70 (0.57)^B^	30.69 (0.66)^B^	36.37 (0.33)^C^
5Y‐MZ‐1	29.14 (1.69)^C^	32.02 (0.74)^D^	35.04 (1.05)^C^	39.22 (0.36)^C^	45.75 (0.84)^D^
5Y‐MZ‐2	27.48 (1.74)^C^	30.63 (1.19)^D^	34.15 (0.76)^C^	37.50 (0.78)^C^	42.40 (0.70)^E^

*Note:* Mean values are presented with their respective standard deviation in parentheses. Different superscript letters indicate significant differences between the respective materials. Statistical significance was assessed separately for each thickness.

**TABLE 3:** Mean translucency parameter of the investigated materials across different wall thicknesses calculated using CIEDE2000.

Material	Specimen thickness [mm]				
	1.25	1.00	0.75	0.50	0.25
3Y‐PZ	7.48 (0.19)^A^	8.67 (0.14)^A^	10.01 (0.30)^A^	13.43 (0.40)^A^	17.98 (0.70)^A^
5Y‐PZ	13.34 (0.24)^B^	15.03 (0.41)^B^	17.27 (0.54)^B^	19.68 (1.06)^B^	22.57 (1.18)^B^
3Y‐MZ	14.25 (1.29)^B^	16.43 (0.61)^C^	18.27 (0.54)^B^	20.54 (0.50)^B^	24.83 (0.25)^C^
5Y‐MZ‐1	20.04 (1.21)^C^	22.03 (0.42)^D^	24.25 (0.57)^C^	27.35 (0.37)^C^	33.00 (0.80)^D^
5Y‐MZ‐2	18.54 (1.23)^D^	21.06 (0.89)^D^	23.76 (0.59)^C^	26.61 (0.56)^C^	31.04 (0.49)^E^

*Note:* Mean values are presented with their respective standard deviation in parentheses. Different superscript letters indicate significant differences between the respective materials. Statistical significance was assessed separately for each thickness.

When comparing the TP of materials with similar Y_2_O_3_ contents, 3D‐printed zirconia showed significantly lower values than their milled counterparts. For the comparison of 3Y‐PZ and 3Y‐MZ, this was reflected by an average TP_*ab*_ difference of 10.56 across all thicknesses, whereas average TP_*ab*_ for 5Y‐MZ‐1 and 5Y‐MZ‐2 surpassed those of 5Y‐PZ by 9.80 and 8.00, respectively. Moreover, all differences between the 3Y‐PZ and 3Y‐MZ groups, as well as between the 5Y‐PZ and 5Y‐MZ‐1 or 5Y‐MZ‐2 groups, were statistically significant.

Overall, 3Y‐PZ had the lowest TP, followed by 5Y‐PZ, 3Y‐MZ, 5Y‐MZ‐2, and 5Y‐MZ‐1, with that of 5Y‐PZ in the same range as that of 3Y‐MZ.

### 3.2 Microstructural Analysis

**FIGURE 4:**
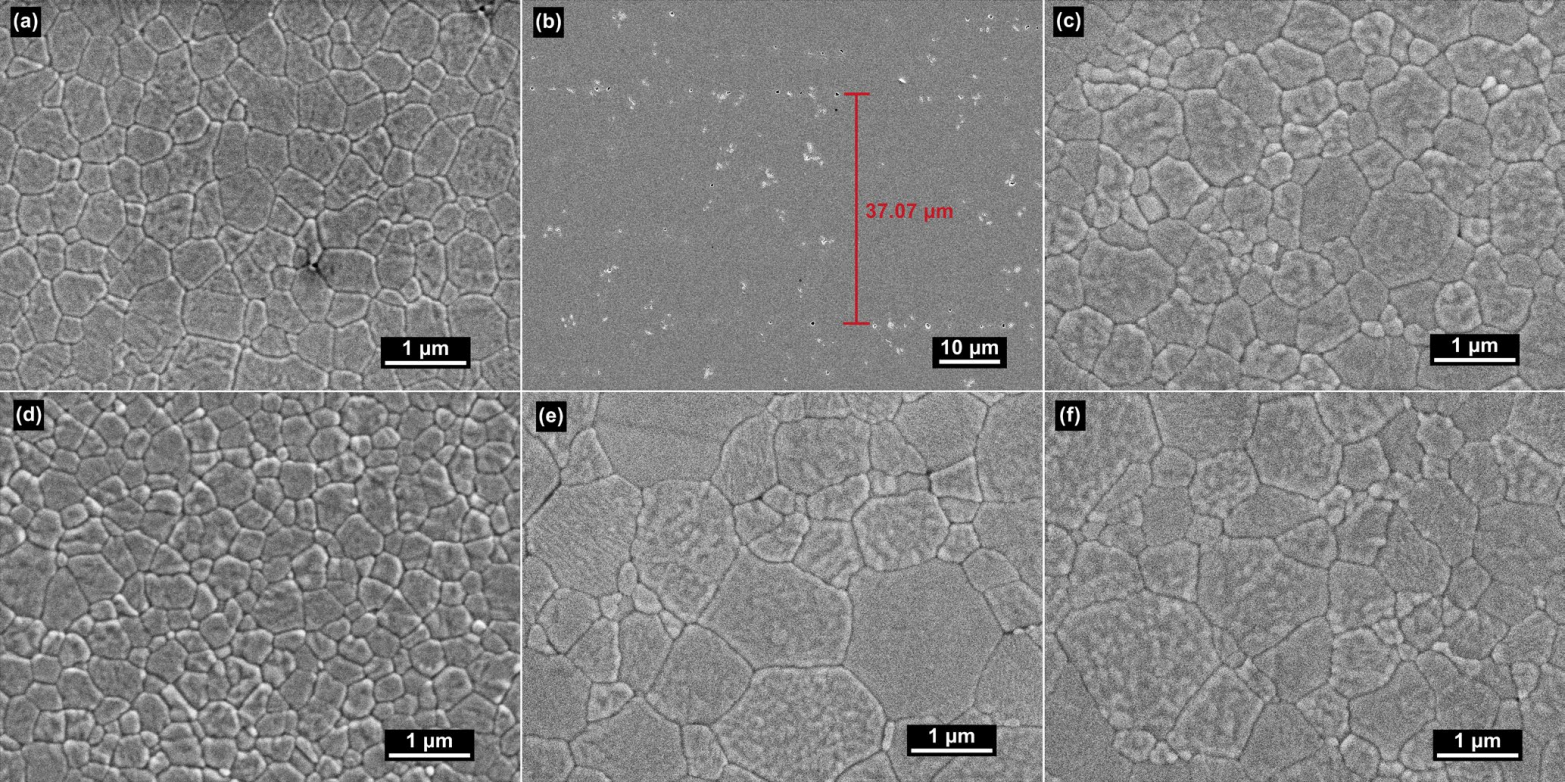
Scanning electron microscope (SEM) images of the five materials. (a) Image of 3Y‐PZ at 20,000× magnification. (b) Sectioned 3Y‐PZ sample at 1500× magnification revealing a microporosity predominantly located at printing layer interfaces with a measured distance of 37.07 μm, closely aligning with the calculated target value of 36.66 μm. This target value was derived by dividing the layer height during printing (50 μm) by the scaling factor (1.3639). (c–f) SEM images of (c) 5Y‐PZ, (d) 3Y‐MZ, (e) 5Y‐MZ‐1, and (f) 5Y‐MZ‐2 at 20,000× magnification.

The mean grain size differed significantly between the groups based on microstructural analysis (Table 4). As expected, the 3Y‐TZP materials (3Y‐PZ and 3Y‐MZ) had smaller mean grain sizes than the 5Y‐PSZ materials (5Y‐PZ, 5Y‐MZ‐1, and 5Y‐MZ‐2). Overall, 3Y‐MZ exhibited the smallest grain size, followed by 3Y‐PZ, 5Y‐PZ, 5Y‐MZ‐2, and 5Y‐MZ‐1. Representative images of all the materials are shown in Figure 4a,c–f, which illustrate the differences in grain size among the materials.

**TABLE 4:** Mean grain size of the investigated materials.

Material	Mean grain size [μm] (SD)
3Y‐PZ	0.513 (0.027)^A^
5Y‐PZ	0.744 (0.024)^B^
3Y‐MZ	0.461 (0.007)^A^
5Y‐MZ‐1	1.023 (0.082)^C^
5Y‐MZ‐2	0.820 (0.042)^B^

*Note:* Different superscript letters indicate significant differences between the respective materials.

Microstructural analysis revealed a higher amount of microporosity (pore diameters were in general < 1 μm) in both 3D‐printed zirconia materials (compared to milled counterparts) that accumulated predominantly along the interfaces between the individual printing layers (50 μm layer thickness during printing was reduced to about 37 μm due to sintering shrinkage) as shown in Figure 4b.

## 4 Discussion

The materials under investigation showed distinct differences in their translucency. The 3D‐printed materials exhibited significantly lower TP values than their milled counterparts with the same yttria dotation. Therefore, the first hypothesis is accepted. Microstructural differences between the respective materials were most likely the cause of these differences in the TP. First, smaller grain sizes lead to lower translucency due to the increased number of grain boundaries in the microstructure [5, 30, 31, 32]. Light is scattered at the grain boundaries because of differences in crystallographic order, refractive index, and potential defects. This partially explains the lower TP value of 5Y‐PZ compared to its two milled counterparts. Second, the higher number of pores observed in the printed materials substantially decreases their translucency. Similar to the grain boundaries, this was due to light scattering caused by the disparity in refractive indices between air and zirconia [21]. While the mean grain size of 3Y‐PZ was larger than 3Y‐MZ, the scattering effect of the pores predominantly determined the lower TP of the 3D‐printed specimen. All differences between the 3D‐printed and milled materials of the same yttria dotation (mean ΔTP_*ab*_ of 10.56 between 3Y‐PZ and 3Y‐MZ and mean ΔTP_ab_ of 9.80 and 8.00 between 5Y‐PZ, 5Y‐MZ‐1 and 5Y‐MZ‐2, respectively) are not only significant, but clearly exceed the translucency acceptability threshold (TAT) of ΔTP_*ab*_ = 4.43 [33]. The same applies to the TP values, calculated using CIEDE2000 and the respective TAT of ΔTP_00_ = 2.62. Merely based on the translucency of the 3D‐printed 3Y‐TZP materials, they therefore are not a clinically acceptable alternative for each indication to their more established milled counterparts.

Within each manufacturing group (3D‐printing and milling), the expected trend of higher amounts of yttria and larger grain sizes resulted in higher TP values. Therefore, the second hypothesis is accepted. In addition to the reduced number of grain boundaries, higher yttria doping (in this case, up to 5 mol%) led to the partial stabilization of the cubic phase alongside the tetragonal phase. As the cubic phase is optically isotropic, meaning it has the same refractive index in all directions, light scattering at the grain boundaries is further reduced. The higher translucency with increased yttria doping is reflected in significantly higher TP_*ab*_/TP_00_ values that both exceed the translucency perceptibility threshold (TPT) (1.33 and 0.62 corresponding to CIELAB and CIEDE2000, respectively [33]) and TAT at all thicknesses.

The measured translucency values for MZ specimens are within the range of values, although predominantly higher, than that of previous studies [7, 34, 35, 36]. Variations in the measuring equipment, specimen preparation, and reference background are possible explanations. Differences in the methods used to assess a material's translucency and the variability within each of these methods reported in the literature make a quantitative comparison with the indications provided by manufacturers difficult.

In this study, the TP was assessed using the two most common forms of calculation (CIELAB/TP_*ab*_ and CIEDE2000/TP_00_) [33, 37]. Although, CIELAB is the more widely used formula in literature and therefore enables easier comparison to related studies, CIEDE2000 has a better fit with the visual judgment and is currently recommended by the International Commission on Illumination (CIE). The different calculations do not affect the overall effects that are discussed in this study, with the only exception being that the ΔTP_00_ between 5Y‐MZ‐1 and 5Y‐MZ‐2 at 1.25 mm thickness turned out not to be significant with the chosen level of significance of *α* = 0.05.

High translucent variants of 3Y‐TZP materials used for milling, such as the one investigated in this study (3Y‐MZ), are well established for monolithic restorations due to their high mechanical strength, biocompatibility and sufficient translucency for use in posterior regions [32, 38, 39, 40, 41, 42]. With flexural strengths that are considerably higher than 500 MPa, the printed 5Y‐PSZ material examined in this study has been shown to meet the requirements for single crowns and three‐unit fixed partial dentures according to ISO 6872 (class 5) [19]. Since no statistically significant differences in TP_*ab*_/TP_00_ between 5Y‐PZ and 3Y‐MZ were observed for thicknesses of 1.25, 0.75 and 0.50 mm and the differences for 1.00 and 0.25 mm are below their respective TAT, their translucency is comparable. Based on its flexural strength and translucency, the clinical use of 5Y‐PZ is justified. The impact of material thickness is also in accordance with previous studies which investigated the translucency of milled zirconia materials [12].

The influence of heat treatment duration and material thicknesses on porosity formation and translucency remains a subject for future investigation. It is likely that faster heat treatments lead to increased porosity, as insufficient time for complete binder removal results in the formation of residual pores or cracks during debinding. Moreover, shortening the sintering time decreases the grain size and further reduces translucency [43, 44]. In this study, a conservative debinding and sintering protocol was applied in relation to the manufactured sample thicknesses.

Currently, there is limited research available on the translucency of 3D‐printed zirconia [9, 21, 24, 28, 45]. To date, no studies have specifically investigated the material evaluated in the present study. In accordance with the findings of this study, the main challenge is to reduce the porosity and defects of the specimens to increase their translucency.

To this end, a unique strength of 3D‐printing is the fabrication of thin and defect‐oriented restorations. Given its increased translucency, the ability to maintain thinner wall thicknesses and its superior mechanical strength compared to glass ceramics like lithium disilicate, 3D‐printed 5Y‐PSZ is recommended for this indication. For other indications, especially in the anterior region no advantages over the milled counterparts—if fabrication is possible—are rendered at the moment. Generally, high‐end esthetical demands in the anterior region require additional labial veneering.

## 5 Conclusions

Within the limitations of this current study, it was concluded that microstructural differences cause decreased translucency in 3D‐printed zirconia compared to milled counterparts. Nonetheless, the translucency of 3D‐printed 5Y partially stabilized zirconia is sufficient for monolithic restorations in the posterior region, since it is comparable with high translucent milled 3Y tetragonal zirconia polycrystal materials.

## Funding

This work was supported by Deutsche Forschungsgemeinschaft, 524875261.

## Ethics Statement

The authors have nothing to report.

## Conflicts of Interest

The authors declare no conflicts of interest.

## Data Availability

The data that support the findings of this study are available from the corresponding author upon reasonable request.
